# Epithelial maturation and molecular biology of oral HPV

**DOI:** 10.1186/1750-9378-4-16

**Published:** 2009-11-25

**Authors:** Liviu Feller, Razia AG Khammissa, Neil H Wood, Johan Lemmer

**Affiliations:** 1 Department of Periodontology and Oral Medicine, School of Dentistry, University of Limpopo, Medunsa Campus, South Africa; 2 Professor Emeritus, Department of Oral Medicine and Periodontology, School of Dentistry, University of the Witwatersrand, Johannesburg, South Africa

## Abstract

Human papillomavirus (HPV) is widespread and can cause latent infection in basal cells, with low HPV DNA copy-number insufficient for transmission of infection; can cause subclinical infection that is active but without clinical signs; or can cause clinical infection leading to benign, potentially malignant or malignant lesions. The HPV cycle is influenced by the stage of maturation of the infected keratinocytes, and the production of virions is restricted to the post-mitotic suprabasal epithelial cells where all the virus genes are expressed.

Low-risk HPV genotypes are associated with the development of benign oral lesions, whereas high-risk HPV genotypes are implicated in the development of malignant epithelial neoplasms. The rôle of high-risk HPV as a causative agent in epithelial malignancy is different at different anatomical sites: it is almost invariably implicated in squamous cell carcinoma of the uterine cervix, fairly frequently implicated in squamous cell carcinoma of the oropharynx, and it is seldom implicated in squamous cell carcinoma of the mouth.

## Introduction

Human papilloma viruses (HPV) are members of the papillomaviridae family that infect epithelial cells exclusively. After gaining entry into the cells of the epithelial basal layer, replication of the virus occurs in the nuclei of the infected cells, and the production of mature virions occurs in the suprabasal epithelial cell layers [1]. HPV infection is highly transmissible, has a variable incubation period that can culminate in latent infection with low HPV DNA copy-number in basal cells insufficient to support transmissibility; in subclinical infection that is active but without clinical signs; or in clinical infection leading to benign, potentially malignant or malignant epithelial lesions. Many of these manifestations of HPV infection can undergo spontaneous resolution [2].

HPV infection is the most common of all sexually transmitted diseases. It is estimated that two thirds of those who have had sexual contact with HPV-infected persons, will become infected [3]. Oral HPV infection can be acquired by oral-genital contact, by mouth-to-mouth contact, or possibly by autoinoculation [4,5]; and in infants by mother-to-child transmission [6,7]. How HPV infection of the upper respiratory tract occurs is not clear, but it may be by mucous carriage of virally infected squames from the mouth, or from the mouth of another person to the oropharynx and larynx [8].

The clinical manifestations and the microscopical features of HPV-associated lesions vary with the anatomical site affected and with the genotype of the HPV [7]. The variability of the clinical and microscopical appearances and the course of HPV infection are governed by the complex interactions between the specific HPV genotype, viral genetic variables, host immune response and the phenotype of the infected epithelial cell against a background of differing environments and life-styles [9].

Immunosuppressed people are at significantly greater risk of developing HPV infection and of experiencing a more aggressive course of infection than immunocompetent people. The immune system plays an important rôle in controlling HPV infection [2,10], but since the intracellular HPV is shielded from the host immune surveillance until the virus has been sufficiently amplified or the infected keratinocytes exfoliate and disintegrate, the immune response remains relatively low-level compared to the immune response to viruses that are not confined to epithelial cells [10,11].

More than 100 types of HPV have been identified, differing in the regulatory sequences and coding potential of their genomes [9,10,12]. Because of the unequivocal implication of HPV in the aetiopathogenesis of uterine cervical squamous cell carcinoma (SCC), HPV types in this context have been well studied and categorized into low-risk and high-risk types according to their potential for causing SCC [13]. Examples of low-risk HPV genotypes are HPV-6, 11, 42, 43, 44 and of high-risk HPV genotypes are HPV-16, 18, 31, 33, 35, 45, 51, 52, 56, 58 and 59 [6]. Distinct clinical manifestations are associated with specific HPV genotypes [2,6,13-18] (Table 1).

**Table 1:** HPV genotypes and their associated diseases.

Diseases and anatomical sites	HPV genotype	References
1. Benign oral lesions		
1.1 Oral squamous cell papilloma	HPV types 6 and 11	[16,17]
1.2 Veruca vulgaris (common wart)	HPV types 1, 2, 4, 7 and 57	[16,17]
1.3 Condyloma acuminatum	HPV types 2, 6, 11 (and less frequently HPV types 16, 18, 31, 33 and 35	[2,16-18]
1.4 Focal epithelial hyperplasia (Heck disease)	HPV types 13 and 32	[2,16-18]
2. Potentially malignant oral lesions		
2.1 Leukoplakia	HPV types 16 and 18	[27-29]
	HPV types 6 and 11	[25,28]
2.2 Erythroplakia	HPV types 6, 11, 18, 31 and 33	[25,28]
3. Oral and oropharyngeal squamous cell carcinoma	HPV types 16 and 18	[14-18]
4. Recurrent respiratory papillomatosis	HPV types 6 and 11	[2,16-18]
5. Anogenital		
5.1 Condyloma acuminata	HPV types 6 and 11	[2,13]
5.2 Intraepithelial neoplasia		
5.2.1 Low-grade	HPV types 6 and 11 (less frequently HPV types 16, 18, 31, 33, 35)	[2,13]
5.2.2 High-grade	HPV types 16 and 18 (less frequently HPV types 6, 11, 31, 35)	[2,13]
5.3 Squamous cell carcinoma	HPV types 16 and 18 (less frequently 31, 33, 35, 39, 45, 51, 52, 58)	[2,6,13]
6. Cutanous		
6.1 Common warts	HPV types 1 and 2	[2]
6.2 Flat warts	HPV types 3 and 10	[2]

In a small minority of women with persistent vulvo-vaginal infection with high-risk HPV genotypes, cervical SCC may develop as long as 12-15 years after the initial infection [12]. High-risk HPV genotypes, in particular HPV-16, have also been implicated in the development of oral and oropharyngeal SCC [14,15,19-24].

Low-risk HPV genotypes only rarely have been implicated in malignant transformation [6]. In the mouth, low-risk HPV infection is associated with a variety of benign lesions including squamous cell papilloma, verruca vulgaris (common wart), condyloma acuminatum and focal epithelial hyperplasia (Heck disease), but can also be detected in potentially malignant epithelial lesions such as leukoplakia and erythroplakia [25,26].

High-risk HPV genotypes, in particular HPV-16 and -18 are prevalent in potentially malignant oral epithelial lesions [27-29] and in oral SCC [30,31]; but the cells of these lesions typically show low viral load, infrequent viral integration into the cellular genome, and the transformed cells seldom contain active transcriptional E6/E7 mRNA [31-33].

## HPV Genome Organization and the Function of some of the HPV Gene Proteins

**Figure 1:**
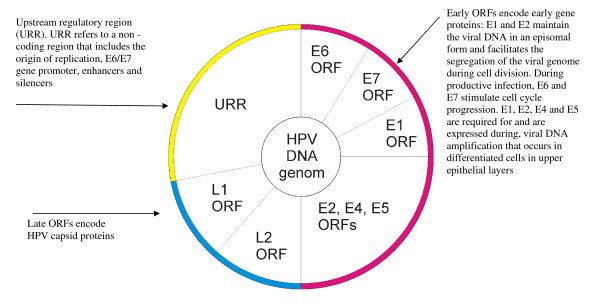
**The circular organization of HPV DNA episome (adapted from references **[2,10,22]**)**.

All types of HPV have the same general organization of the genome: double-stranded circular DNA that carries early (E) open reading frames (ORFs) encoding for non-structural regulatory proteins, and late (L) ORFs encoding for capsid proteins. Noncoding upstream regulatory region (URR) encompassing the origin of replication, the E6/E7 gene promoter, and enhancers and silencers, is located between the early and the late regions (Figure 1) [2,3].

Early ORFs encode for E1, E2, E4, E5, E6 and E7 proteins. E1 and E2 proteins bind to viral origin of replication, recruit cellular proteins that mediate viral DNA replication, and regulate viral gene expression [3,7]. E1 and E2 are also responsible for the maintenance of viral DNA as an episome during initial HPV infection, and also during latent infection in the basal cell layer [3,10,34]. E2 facilitates the segregation of the HPV genome during cell division resulting in the distribution of HPV DNA into daughter cells [10,34]; and is involved in the packaging of HPV DNA and in the promotion of virion assembly. Furthermore, E2 has the capacity to repress the activity of E6/E7 promoter [34].

The integration of HPV DNA within the cellular genome that is evident in some HPV-associated malignancies disrupts the E2 ORF leading to loss of E2 repressing function [35], thus permitting free transactivation of E6/E7 promoter by cellular transcription factors [2,11] and possibly by other factors such as nutritional agents, resulting in the increased expression of E6 and E7 oncoproteins [34].

E3 ORF does not encode for proteins [2]. The function of E4 and E5 HPV proteins is not completely known [3]. E4 protein is assumed to modulate HPV DNA amplification and structural gene expression [10,34]. E5 protein participates in HPV DNA amplification. It is also associated with down-regulation of gap junction intercellular communications, with expression of major histocompatibility complex (MHC) class 1 molecules, and with activation of growth factor receptors. Thus E5 protein has the capacity to promote immune evasion [34,36].

Little is known about the action of E6 and E7 proteins of low-risk HPV genotypes during latent and subclinical HPV infection [2,3]. During productive HPV infection high- and low-risk HPV E6 and E7 facilitate the maintenance of the viral episome in suprabasal matured epithelial cells [3]. E6 and E7 also play an important rôle in the increased cell proliferation and the extended cell survival of HPV-associated malignancies, by altering the cell cycle regulatory factors [36,37]. E6 of high-risk HPV can induce ubiquitin-mediated degradation of p53 tumour suppressor protein [38,39]; and E7 of high-risk HPV genotypes can bind to and inactivate the Rb suppressor gene and other cellular proteins associated with cell cycle regulation [39,40]. Consequently E6 and E7 of high-risk HPV genotypes are instrumental in the pathogenesis of HPV-associated malignancies.

E6 and E7 of low-risk HPV probably play a rôle in the pathogenesis of HPV-associated benign tumours and only rarely are implicated in malignant transformation. It is unclear what properties E6 and E7 of high-risk HPV genotypes possess that the E6 and E7 of low-risk HPV genotypes do not possess, that enable them to induce malignant transformation of some epithelial cells infected with high-risk HPV. One possibility is that E6 and E7 proteins of high-risk HPV genotypes have a greater affinity for the regulatory factors of the cell cycle, thus causing an exaggerated dysregulation of mechanisms of cell growth and of cell survival [2].

E6 and E7 are not expressed in cells of the basal layer with low-risk HPV infection [2], and it is uncertain whether E6 and E7 oncoproteins of high-risk HPV genotypes are expressed in basal cells during productive HPV infection that does not give rise to malignant changes [10].

In high-risk HPV infection associated with intraepithelial neoplasia or with invasive malignancy, E6 and E7 oncoproteins are expressed in progenitor basal layer keratinocytes mediating basal cell proliferation and genomic instability, making the infected cells susceptible to additional genetic alterations leading to cell transformation and possibly to the subsequent development of SCC [35]. The biological effects induced by E6 and E7 of high-risk HPV have been investigated in great detail. Little is known about the biological properties and mode of action of E6 and E7 proteins of low-risk HPV genotypes [2].

E8 ORF has been identified only in bovine papillomavirus [2]. L1 ORF encodes for major capsid protein and L2 ORF for minor capsid protein [2]. L1 protein is expressed after L2 protein in the viral replication cycle and mediates packaging of HPV particles and virion assembly. L2 protein interacts with E2 protein, facilitates the transport of L1 protein to the nucleus, and plays a role in the encapsulation of viral DNA [10].

## Epithelial Cell Proliferation and Maturation

The cells of the epithelium comprise two populations of cells, the progenitor and the maturing. The former cells are based in the basal and parabasal layers, have the capacity to proliferate by mitotic division following which some of the newly divided (amplifying) cells enter a maturation process and gradually rise through the several morphologically distinct cell layers to the surface, while some remain to maintain the integrity of the basal layer. In the mouth this process of maturation follows two main patterns: keratinization, and non-keratinization [41].

Some authors refer to this process of maturation as differentiation [2,3,10]. The authors of this paper prefer the term maturation, as we think that the term differentiation should be restricted to the development of specific populations of stem cells arising from the zygote that form particular cell compartments and possess distinct characteristics such as function, shape and rate of turnover [42].

Whether the cells that give rise to new progenitor cells and those that give rise to cells destined to go through the process of maturation are in fact distinct sub-populations, or whether the fates of all cells resulting from mitoses in the basal cell layer are determined by changing local microenvironment, is uncertain.

## HPV Cycle as it Relates to Epithelial Cell Maturation

The HPV cycle is influenced by the stage of maturation of the infected keratinocyte in the squamous epithelium [2,3,10]. The production of virions is restricted to mature suprabasal epithelial cells where all the virus genes are expressed [43]. Why HPV virions can be produced only by suprabasal postmitotic infected epithelial cells is not known [3].

HPV infection starts when the viruses enter epithelial basal cells which are referred to as the target cells of the virus [10,44]. Once inside the target cell, the viral DNA undergoes uncoating and is transported to the nucleus where the HPV genome will persist as multiple episomal copies [10]. In HPV-infected basal cells, E1 and E2 proteins are expressed and they regulate early viral DNA transcription from the early promoter prior to production of virions. When expression of E2 is more pronounced, E2 represses viral DNA replication by blocking cellular transcription factors, thus controlling the number of HPV DNA copies in the basal cell by a process analogous to negative feedback [3]. A stable number of about twenty to one hundred HPV DNA copies per cell is regulated by E1 and E2 viral proteins in the basal cells for as long as the infection persists [3].

In cases of high-risk HPV infection, E6 and E7 may also be expressed in HPV-infected basal cells, and the epithelium may then enter a proliferative phase characterized by an increasing number of HPV-infected basal cells [10], culminating in intraepithelial or invasive neoplasm.

Why HPV can infect basal cells in the first instance but not the cells of other epithelial layers is not known; but this is probably related to specific surface receptors confined to basal cells [2]. The attachment of the virus to the basal cell membrane receptor may be mediated by heparin sulphate, and the bound virus enters the cell by a slow process of endocytosis [10]. As the basal cells divide, E2 mediates distribution of some HPV DNA copies to daughter cells, while some copies remain in the progenitor cells, in both cases, as episomes [35].

In latent HPV infection the affected epithelium looks both clinically and histologically normal because the gene expression of the latent genes is restricted to E1 and E2 [10]. The virus can remain latent for long periods, can be cleared by the immune system, or can be activated and initiate productive infection [35].

As the epithelial cells mature, the HPV cycle progresses to productive replication [10,43]. In HPV infected epithelium where virions are being produced the matured epithelial cells express HPV E6 and E7 proteins in the suprabasal layers. E6 prevents apoptosis that normally occurs in response to viral infection; and E7 has the capacity to overcome the physiological blockade of DNA synthesis at the G1/S phase of the cell cycle in these post-mitotic cells. Thus E7 activates the cellular DNA replication mechanism allowing matured epithelial cells to re-enter the S phase of the cell cycle, and although this does not usually result in full replication of the cellular genome it makes the cellular replication machinery available for viral DNA replication [3,35,37,45].

Multiplication of the viral genome, synthesis of early gene proteins (E2, E4, E6, E7) and late gene proteins (L1, L2), assembly of virions, and release of virions from exfoliated epithelial cells occur at progressive stages of maturation of postmitotic HPV-infected epithelial cells. It is suggested that specific cellular factors associated with epithelial cell maturation activate late viral promoter located within E7 ORF resulting in expression of high levels of E1 and E2 viral replication proteins which activate late viral gene expression. This increase in E1 and E2 expression leads to HPV amplification resulting in an increase in viral genome numbers parallel with the stages of epithelial cell maturation. Eventually, mediated by L1 and L2 proteins, the virus escapes from the shedding epithelial cells [10,39].

Unlike the early promoter, the late promoter is not suppressed by high levels of E2 [3]. The amplification of HPV genome in the maturing epithelium is modulated by E4 and E5 proteins, and E6 and E7 proteins ensure the maintenance of the HPV genome as a stable replicating episome. As outlined above, under certain circumstances E6 and E7 have the capacity to stimulate suprabasal postmitotic cells to re-enter the S-phase of the cell cycle resulting in the development of proliferative epithelial lesions [3]. However, during subclinical HPV infection, although viral production continues, for as yet unknown reasons, the ability of E6/E7 to mediate the re-entry of suprabasal post-mitotic epithelial cells at the S phase of the cell cycle is limited, suprabasal proliferation of epithelial cells does not occur, and proliferative epithelial lesions do not develop [10]. In some instances, normal appearing epithelial cells with subclinical infection may be adjacent to the HPV-induced lesion [7].

HPV-induced benign lesions are characterized by proliferation of cells of all epithelial layers [46]. This proliferation is most probably mediated by E6/E7 proteins of mainly low-risk HPV. The proliferation manifests microscopically as acanthosis, hyperkeratosis and parakeratosis [7,46]. Koilocytes in the upper layers of the epithelium are evident [7].

Microscopic examination of high-risk HPV-induced high-grade intraepithelial neoplasia shows greatly increased numbers of mitotic figures and features of cellular atypia, and extension of these multiplying atypical cells into the suprabasal layer of the epithelium as the outcome of expression of high-risk HPV E6/E7 oncoproteins in the basal cells [35].

## HPV-Associated Oral Benign, Potentially Malignant and Malignant Oral Lesions

In persons who have been exposed to HPV, several HPV types have been detected in their apparently normal oral mucosa, and in certain specific benign, potentially malignant and malignant oral lesions, in the aetiology of which they appear to be implicated. In infected persons, in clinically and histologically normal looking oral mucosa, the HPV exists in a latent form as a low-copy number episome in basal epithelial cells [17]. In HPV-associated lesions, there is a substantial increase in intraepithelial HPV DNA copy-numbers [39].

Oral HPV infection is acquired primarily by sexual transmission, or less frequently by non-sexual direct transmission, by mother to child transmission or by autoinoculation. Persons who practise oral-genital sex, those who have had a number of sexual partners, and those who are immunocompromised are at greater risk of acquiring oral HPV infection [4,5,14,47-49].

### HPV-associated benign oral lesions

Many of the low-risk genotypes and uncommonly, high-risk types have been found in the HPV-associated benign oral lesions, oral squamous cell papilloma, verruca vulgaris (common wart), condyloma acuminatum and focal epithelial hyperplasia (Heck disease) [17,18,50,51], collectively termed oral warts. The four types of oral warts share the characteristics of being exophytic, sessile or pedunculated, or of having filiform or 'cauliflower'-like surface. The lesions can be single, multiple, or clustered [17,49,52], are usually painless and chronic, and occasionally regress spontaneously [53].

Although certain HPV types have been implicated in the aetiology of the different oral warts, there is considerable overlapping of viral types among the lesions, and not infrequently viral types not generally associated with a particular lesion, can be identified. Therefore viral typing, which one would expect to be a diagnostic differentiator of oral warts cannot always be relied upon.

### HPV-associated potentially premalignant and malignant lesions

The reported rates of HPV DNA detection in potentially malignant (leukoplakia, erythroplakia) and malignant (squamous cell carcinoma) oral lesions range from 0 to 100% [54,55]. This extreme variation is owing to differences in ethnicity, geographic locations and sample size of the subjects examined, and to variations in methods of detection of HPV.

High-risk HPV genotypes, in particular HPV-16 and -18, have been reported to be the most prevalent in oral squamous cell carcinoma [30,31,56-58], and HPV-16 in oral leukoplakia including proliferative verucous leukoplakia [27,28]. However, there are reports that low-risk rather than high-risk HPV genotypes are more prevalent in oral leukoplakia [23,25]; and co-infection with several different HPV-genotypes is not uncommon [28,59].

In a meta-analysis of data from 94 studies of 4580 specimens, Miller and Johnston (2001) determined that the likelihood of HPV to be detected in normal oral mucosa, in non-dysplastic leukoplakia, in dysplastic oral leukoplakia and in other precancerous intra-epithelial oral neoplasms, and in oral squamous cell carcinoma is likely to be about 10%, 20%, 26% and 47% respectively. This suggests that HPV may have an oncogenic or a co-oncogenic rôle in some HPV-cytopositive potentially malignant and malignant oral epithelial neoplasms [23].

With HPV-16 cytopostive oropharyngeal SCC as a model of HPV-mediated epithelial cancerization, a causal association between HPV and oral SCC is likely if the cells of HPV-cytopositive oral SCC express transcriptionally active E6/E7 mRNA, frequently demonstrate viral integration within the cellular genome, show high viral load (> 1 copy per cell), show unmutated p53 gene, reduced expression of Rb proteins, overexpression of p16^INK4A^, and infrequent loss of heterozygosity (LoH) at chromosomal loci 3p, 9p and 17p [33,60-66].

As the cells of HPV-cytopositive oral squamous cell carcinoma and potentially malignant lesions are typically characterised by infrequent expression of transcriptionally active E6/E7 mRNA, by infrequent viral integration within the cellular genome, by low HPV viral load and by frequent LoH at chromosomal loci 3p, 9p and 7p [32,33,66]; and as overexpression of p16^INK4A^, of p53 and of Rb proteins is not unequivocally associated with the presence of high-risk HPV DNA [31], there would appear to be only a weak causal association between the HPV in the cells and the process of malignant transformation.

It is possible that HPV super-infection of initially transformed oral keratinocytes may promote progression of transformation in an additive or synergistic manner, through mechanisms as yet unknown [62], or that in some HPV-cytopositive transformed keratinocytes that do not express E6/E7 mRNA, E6/E7 oncoproteins have participated in the initial stages of transformation but phased out during later stages [67].

Although it is clear that there is some link between HPV and potentially malignant and malignant epithelial oral lesions, the precise rôle of the virus in the aetiopathogenesis of these oral lesions is unknown.

## Summary

**Figure 2:**
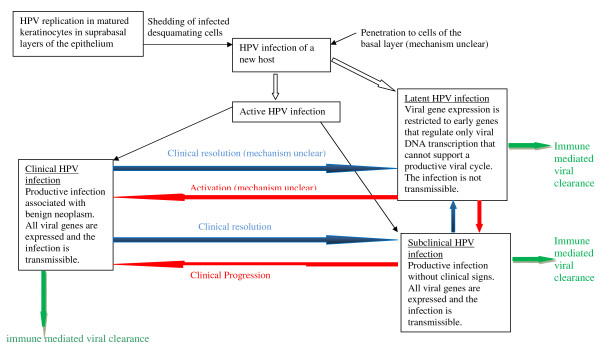
**The manifestations of HPV infection and their interrelationships**. Any of these manifestations can undergo spontaneous resolution. This diagram does not reflect the potentially malignant/malignant related aspects of HPV infection.

HPV is epitheliotropic, and it may be present in a latent form, a subclinical form or in a form which in association with other ill-defined factors can induce benign or malignant epithelial neoplasms. Figure 2 summarizes the different manifestations of HPV infection and the relationships between latent, subclinical and clinical HPV infection.

While some viral proteins are expressed in HPV-infected basal cells independently of the cell maturation process regulating early viral DNA transcription from early promotor prior to production of virions, the productive phase of the viral cycle is dependent on specific cellular factors associated with the progressive stages of maturation of post-mitotic cells as the cells gradually rise trough the layers of epithelium to the surface, and exfoliate.

Persistent high-risk HPV infection in a subset of subjects with HPV-cytopositive oropharyngeal epithelium induces genomic instability in these cells, making them susceptible to additional genetic alterations leading to cell transformation and to subsequent development of squamous cell carcinoma. In these oropharyngeal squamous cell carcinomas there is a causal association between HPV infection and the malignancy. In contrast, the nature of the link between high-risk HPV genotypes and potentially malignant and malignant oral epithelial lesions is not known.

Low-risk HPV infection of oral epithelium is associated with squamous cell papilloma, verruca vulgaris (common wart), condyloma acuminatum and focal epithelial hyperplasia (Heck disease); and possibly with the potentially malignant oral epithelial lesions, leukoplakia and erythroplakia.

## Competing interests

The authors declare that they have no competing interests.

## Authors' contributions

LF and RAGK contributed to the literature review. LF, JL and NHW contributed to the conception of the article. LF, JL, NHW and RAG contributed to the manuscript preparation. Each author reviewed the paper for content and contributed to the manuscript. All authors read and approved the final manuscript.
